# Identification of Regional Lymph Node Involvement of Colorectal Cancer by Serum SELDI Proteomic Patterns

**DOI:** 10.1155/2011/784967

**Published:** 2011-12-29

**Authors:** Nai-Jun Fan, Chun-Fang Gao, Xiu-Li Wang

**Affiliations:** Institute of Anal-Colorectal Surgery, No. 150 Hospital of PLA, No. 2 Huaxiaxi Road, LuoYang 471013, China

## Abstract

*Background*. To explore the application of serum proteomic patterns for the preoperative detection of regional lymph node involvement of colorectal cancer (CRC). *Methods*. Serum samples were applied to immobilized metal affinity capture ProteinChip to generate mass spectra by Surface-Enhanced Laser Desorption/ionization Time-of-Flight Mass Spectrometry (SELDI-TOF-MS). Proteomic spectra of serum samples from 70 node-positive CRC patients and 75 age- and gender-matched node-negative CRC patients were employed as a training set, and a classification tree was generated by using Biomarker Pattern Software package. The validity of the classification tree was then challenged with a blind test set including another 65 CRC patients. *Results*. The software identified an average of 46 mass peaks/spectrum and 5 of the identified peaks at m/z 3,104, 3,781, 5,867, 7,970, and 9,290 were used to construct the classification tree. The classification tree separated effectively node-positive CRC patients from node-negative CRC patients, achieving a sensitivity of 94.29% and a specificity of 100.00%. The blind test challenged the model independently with a sensitivity of 91.43% a specificity of 96.67%. *Conclusions*. The results indicate that SELDI-TOF-MS can correctly distinguish node-positive CRC patients from node-negative ones and show great potential for preoperative screening for regional lymph node involvement of CRC.

## 1. Introduction

Pathologic stage represents the most important prognostic factor for patients with colorectal carcinomas (CRCs) [1, 2]. It has been shown that many regional lymph node involvements in CRC are found with the mode of small lymph nodes (less than 5 mm in diameter) [3, 4], and standard pathologic evaluation may overlook these low-volume nodal metastases, thereby failing to identify nodes imperative to accurate staging. Therefore, it is extremely necessary to understand the molecular alterations, which confer a regional lymph node involvement and then use this information to enhance node-staging accuracy and individual patient management.

Because of the marked heterogeneity of CRC, a panel of biomarkers for screening and diagnosis would be most appropriate. Surface-enhanced laser desorption/ionization time-of-flight mass spectrometry (SELDI-TOF-MS), an affinity-based mass spectrometry method using a protein chip modified with a specific chromatographic surface, is a modified matrix-assisted laser desorption/ionization mass system, overcoming many of the limitations of 2-dimensional electrophoresis and matrix-assisted laser desorption/ionization TOF mass spectrometry [5, 6]. This advanced and smart technique's practical application, as to analysis of complex biologic specimens such as cell lysates, serum, and body fluids, can detect multiple protein changes simultaneously with high sensitivity and specificity [5–9]. In recent years, several groups had investigated serum samples from CRC patients and controls to construct patterns for identification of CRC patients from healthy controls with high sensitivity and specificity by using SELDI-TOF-MS and different protein chips [10, 11]. The aim of the current study was to investigate the application of serum SELDI protein profiling for the preoperative detection of regional lymph node involvement state of CRC.

## 2. Matreials and Methods

### 2.1. Patients and Serum Samples

A total of 210 serum samples (including pathologically confirmed node-positive CRC patients and node-negative patients 105 cases) were collected preoperatively from the institute of anal-colorectal surgery of 150th hospital of People's Liberation Army (PLA) from October 2006 to March 2008. The study was approved by the Research Ethics Committee of 150th Hospital PLA, and all CRC patients involved in the study signed an agreement form consenting for the donating their specimens. All patients, diagnosed as colorectal sporadic moderately differentiated adenocarcinoma by postoperative pathology examination, were found to have no evidence of other diseases. The distribution of clinical stages (AJCC, 2004) were as follows: 31 cases were at stage I, 58 stage IIa, 16 stage IIb, 8 stage IIIa, 84 stage IIIb, and 13 stage IIIc (AJCC, 2004). The average age of the Node-positive CRC patients (49 men, 56 women ranging from 31 to 82 years) was 58.4 years and that of node-negative CRC patients (51 men, 54 women ranging from 29 to 78 years) was 56.4 years. The node-positive CRC patients and node-negative patients are age- and gender-matched. Using a case-control study design, the samples were then separated into training set and test set according to the collected date. The training set (including 70 sera from node-positive CRC patients, and 75 from node-negative CRC patients) were collected from October 2006 to September 2007, and the test set (including 35 sera from node-positive CRC patients, and 30 from node-negative CRC patients) were collected from October 2007 to March 2008.

As pooling serum samples may lead to loss of potential biomarkers in SELDI-TOF-MS proteomic profiling [12], quality control sample was offered by a healthy volunteer (male, 42 years old). The quality control serum sample was used to determine reproducibility and treated as a control protein profile for each SELDI-TOF-MS experiment.

Two milliliters of whole blood were collected by venipuncture into a vacuum tube in the morning before food intake (two days before operation) then were deposited to clot at 4°C for 2 hours. The blood was later centrifuged for 20 min at 700 g, aliquoted into 100 *μ*L, and frozen for storage at −80°C until used.

### 2.2. SELDI-TOF-MS Protein Analysis

The IMAC30 array, which is suitable for this work [13], was assembled in 8-well ProteinChip Bioprocessor, pretreated with 50 *μ*L 100 mM CuSO_4_ to each well for 5 minutes at room temperature, washed 5 times with 50 *μ*L distilled water, then incubated with 50 *μ*L neutralizing buffer (100 mM NaAc, pH4) on a platform shaker for 5 minutes, and washed with 50 *μ*L distilled water for 5 times. After being equilibration with binding buffer (100 mmol/L sodium phosphate, 500 mmol/L sodium chloride, pH7.0), the IMAC30 array was chelated with copper for capturing copper-binding proteins through histidine, tryptophan, cysteine, or phosphorylated amino acids. Serum samples were diluted 1 : 3 into U9 buffer (9 M urea, 2%CHAPS, 50 mM Tris-HCl, pH 9.0) and incubated on ice for 30 minutes. Then the diluted samples were diluted 1 : 13 with the U9 buffer. Each array spot was loaded with 50 *μ*L of diluted serum sample with the 8-well ProteinChip Bioprocessor. After incubated in a platform shaker at room temperature for 60 minutes and the unbounded sample being discarded, each spot was equilibrated with the binding buffer (50 *μ*L/spot) twice (5 minutes per time) to remove the nonspecific binding proteins. The array was then quickly rinsed with 150 *μ*L of distilled water before air-drying. Each spot was loaded with 0.5 *μ*L of saturated sinapinic acid solution prepared in 50% (vol/vol) acetonitrile and 0.5% (vol/vol) trifluoroacetic acid. After air-drying, sinapinic acid solution was added again. Then the array was read by ProteinChip reader. Amount of 31 protein chip arrays were done one by one as mentioned above for the analysis of samples. During experiments, the quality control serum sample was used to test the reproducibility of a single IMAC30 chip (intraassay) and that between chips (interassay).

The PBS-II(c) ProteinChip reader was calibrated with the “All-in-one” peptide standard (Ciphergen Biosystems). Each spot was scanned by a laser with the intensity of 200 and a detector with the sensitivity of 9. Mass-to-charge (m/z) ratio was optimized from 2,000 to 20,000, with a maximum of 150,000. The selected sample spots were exposed to the laser beam at 15 different positions, 7 spots for each position. The TOF mass spectra were then collected using Ciphergen's ProteinChip Software 3.1.

### 2.3. Bioinformatics and Biostatistics

The entire dataset was separated into training set and test set before analysis. The training set was used to construct the classification tree, which was consisted of spectra data from 75 node-negative CRC patients and age- and gender-matched 70 node-positive patients. The test set, which was consisted of the other spectra data of 35 node-positive patients and age- and gender-matched 30 node-negative patients, was used to challenge the discriminatory ability of the classification algorithm blindly.

All spectral data were normalized by total ion current after background subtraction. The range of peak masses was settled between m/z 2,000 and 20,000 because the majority of resolved protein/peptides were found in this range. The m/z from 0 to 2,000 was excluded from analysis because they were mainly the signal noises of the energy-absorbing molecule. The Biomarker Wizard Software (Ciphergnen Biosystems) was subsequently used to make peak detection and clustering across all spectra in the training set and test set with the following settings—for peak detection, the signal-to-noise ratio was 3 and minimum peak threshold was 20%, while for cluster completion, the cluster was 0.5% and the signal-to-noise ratio for the second pass was 2. The spectral data were then exported as spreadsheet files. The spectral data of training set were further analyzed by the Biomarker Pattern Software (version 4.0; Ciphergnen Biosystems) to develop a classification tree. The classification tree was set up to divide the training dataset into node-positive CRC patients and node-negative patients through multiple rounds of decision-making in training mode. When the dataset was first transferred to Biomarker Pattern Software, the dataset formed a “root node”. Based on intensity, the software tried to find the best peak to separate this dataset into 2 “child nodes”. To achieve this, the software would identify the peak and set the concerning intensity threshold. If the peak intensity of a blind sample was not higher than the threshold, this peak would go to the left-side child node; otherwise the right-side. The process would go on for each child node until a blind sample entered a terminal node, either labelled as node-positive patients or node-negative patients. Peaks, which were selected during the process to form the model, were the ones that yielded the least classification error when being combined to be used. After cross-validation in test mode, the validity of this decision tree was further verified using the test set data blindly, which is independent of the training set.

### 2.4. Statistical Analysis

Comparison of relative peaks intensity levels between groups was made by using the Student *t*-test and in all cases *P* < 10^−4^ was considered, statistically, significant. Comparison of rates between groups was conducted using the *χ* ^2^ test and *P* < 0.05 was regarded as a significant difference.

## 3. Results

### 3.1. Serum SELDI Profiles of CRC with Regional Lymph Node Involvement versus CRC without Regional Lymph Node Involvement

**Figure 1:**
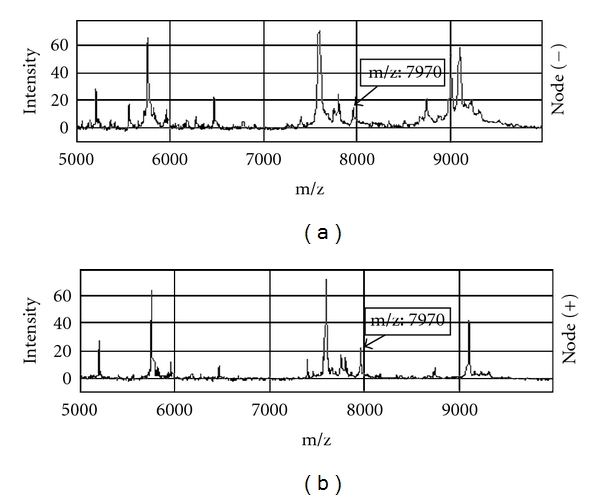
Representative serum protein profiling spectra of node-positive CRC patients and node-negative CRC patients. The peaks at m/z 7,970 were compared. m/z represents the mass to charge ratio.

Spectra from 145 serum samples of CRC patients were acquired in the training set. The protein peaks were identified with masses from m/z 2,000 to 20,000, and 46-peak cluster or common peaks were generated from the identified peaks using the Biomarker Wizard Software. It was found that most of the peaks were detected between m/z 3,000 and 16,500, and the comparability among different samples showed that overall serum profiles from node-positive CRC patients and node-negative patients were very similar despite a few of intersample variations. Therefore, the variations that consistently differentiate these 2 different groups could be considered as the biomarkers of node-positive CRC patients and were considered the most useful for protein profiling. As peaks data from the training set were saved and exported for pattern recognition by Biomarker Pattern Software, a classification tree was created thereby from the training set to discriminate the node-positive CRC patients from node-negative patients.  Figure 1 represents the spectral views showing these protein peaks at m/z 7,970 in these 2 groups. From the quantitative point of view, the average normalized intensities of these proteins were either over- or low expressed in node-positive CRC patients (Table 1). These difference were statistically significant (*P* < 0.05). 

**Figure 2:**
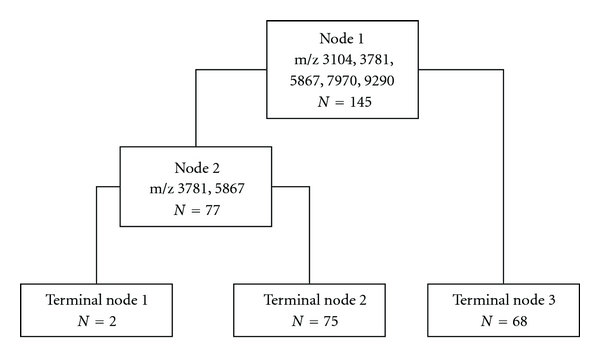
The pattern-matching algorithm to distinguish node-positive CRC patients from node-negative CRC patients on learning mode of training set. The left branch node after Node 1 is the cases of the linear combination: −0.075 (m/z 3,104) −0.231 (m/z 3,781) + 0.157 (m/z 5,867) + 0.086 (m/z 7,970) + 0.953 (m/z 9,290) ≤1.122, and the right one is >1.122. The left branch node after the Node 2 is the linear combination: 0.410 (m/z 3,781) −0.912 (m/z 5,867) ≤0.248, and the right one is >0.248. The cases met to the conditions of Terminal Node 1 and Terminal Node 3 were diagnosed as node-positive CRC patients, and those met to the conditions of Terminal Node 2 were diagnosed as node-negative CRC patients. *N* represents the number of samples. m/z represents the mass to charge ratio.

Among these classification trees generated by adjusting the setting of Gini, costs, advance, and testing of Biomarker Pattern software, the optimal classification tree with the lowest error cost was eventually established. The selected classification tree is simple and straightforward and used 2 splitters (Node 1 and Node 2) with distinct masses of m/z 3,104, 3,781, 5,867, 7,970, and 9,290, respectively, and classified 3 terminal nodes (Figure 2). The variable importance score of some peaks were shown in Table 2. The error rate of the generated classification tree was estimated through a process of cross-validation.

Performance of the generated classification tree is summarized for the training and test sets. For training set part, the classification algorithm was firstly challenged on learning mode and achieved an accuracy of 100.00% (145 of 145); secondly it was cross-validated on test mode and achieved an accuracy, sensitivity, and specificity of 97.24% (141 of 145), 94.29% (66 of 70), and 100.00% (75 of 75), respectively. The validity and accuracy of the classification algorithm were then evaluated by challenging to classify blinded objects correctly in the test set. The algorithm correctly classified 93.85% (61 of 65) of the total testing samples with a sensitivity of 91.43% (32 of 35), a specificity of 96.67% (29 of 30), and a positive predictive value of 96.97% (32 of 33) (Table 3).

### 3.2. Quality Control and Reproducibility

The reproducibility of each SELDI protein chip assay spectra, that is, mass and intensity from array to array on a single IMAC30 chip (intra-assay) and between chips (interassay), was determined by the one healthy volunteer serum sample. Three proteins, among the range of m/z 3,000 to 10,000 observed on spectra randomly selected over the course of the study, were used to calculate the coefficient of variance (CV). The intra- and interassay CVs for mass were both 0.03% while the intra- and interassay CVs for the normalized intensity were 17.20% and 19.48%, respectively. Little variations with day-to-day sampling and instrumentation or chip variations were also found.

## 4. Discussion

The causative reason of the death of CRC is associated directly with stage and therapeutic methods. Presence and extent of regional lymph node involvement predict outcome in patients with CRC. In terms of diagnosis, treatment, and survival in patients, completeness of nodal resection and staging accuracy has significant implications with this disease [14]. Up to 30% of patients with node-negative CRC staged by standard pathologic techniques ultimately suffer from disease recurrence and tumor-related mortality following potentially curative primary resection. Traditionally, the methods of local staging for CRC include digital rectal assessment, proctoscopy, transrectal ultrasonography (TRUS), pelvic computed tomography (CT), and magnetic resonance imaging (MRI). More recently, positron emission tomography has also been taken into consideration. In spite of numerous studies and meta-analyses being performed not only for comparing the performance of these staging modalities all together but also for specific parameters (T and N stage, circumferential margin), no general agreement has been achieved [15–17].

The molecular and cellular heterogeneity of CRC results in the expression variety of tumor cell products. Analysis of the resultant protein profile may have greater efficiency in node-staging by selecting a combination of protein alterations (pattern recognition) rather than by focusing on specific tumor marker. Evaluations of conventional serologic markers, such as CEA and CA19-9, have yielded confusing results with poor sensitivity and specificity for use in early detection. To our knowledge, these biomarkers have not yet been used in node-staging.

Two-dimensional gel electrophoresis has traditionally been used to identify differences in protein expression in terms of serum, saliva, or tissue specimens, with identified proteins subsequently being excised from the gel and being subjected to peptide mapping analysis by mass spectrometry, which is used for the identification of proteins [18, 19]. However, it is labor and time intensive that can be hardly to be reproduced. Besides this, its character confines itself for not being able to handle proteins with molecular weights of less than 10 kDa. SELDI-TOF-MS is able to generate high-throughput protein profile that can afterwards be analyzed to tell protein patterns differences between patients with disease and healthy controls; even it can be used to distinguish patients with different disease stage. Proteomic analysis of serum samples from patients with pancreatic, gastric, breast, nasopharyngeal, liver, ovarian, prostate, and colorectal cancer using SELDI-TOF-MS has been approved feasibility to identify reproducible protein profile that is associated with specific tumor biomarkers, which can definitely be used for early detection of disease [5–9].

Currently, we attempt to combine the SELDI protein chip technology and an artificial intelligence classification algorithm to screen serum protein spectra in the Chinese population of node-positive CRC patients and node-negative patients. For the training set, all 145 serum samples were used to profile protein peaks and to detect important peaks. With a panel of 5 peaks, a classification and regression tree was set up by using Biomarker Pattern Software, which yielded a sensitivity of 94.29% and a specificity of 100% in differentiating node-positive CRC patients from node-negative patients. Furthermore, the blind test challenged the model with a sensitivity of 91.43% (32 of 35), a specificity of 96.67% (29 of 30), and a positive predictive value of 96.97% (32 of 33).

Regarding the study of serum biomarkers for CRC, Chen et al. [10] investigated in 55 serum samples from patients with CRC and 92 healthy individuals with corresponding physiological features by using H4 protein chips and SELDI-TOF-MS. The analysis software (artificial neural network classifier) separated CRC from healthy individuals, with a sensitivity of 91% and specificity of 93%. Four top-scored peaks at m/z of 5,911, 8,930, 8,817, and 4,476, were finally selected as the potential “fingerprints” for detection of CRC. Liu et al. [11] used SELDI protein chip (IMAC3) arrays to screen both patients with CRC and health people. The Biomarker Wizard and Biomarker Pattern Software packages were also applied and then constructed a pattern with 2 protein peaks, achieving a sensitivity of 95.00% and specificity of 94.87%, respectively, in masked analysis of an independent set of serum samples. As for staging of CRC, Xu et al. [20] detected the serum proteomic pattern by using SELDI-TOF-MS technology and CM10 protein chip in CRC. They built up a model formed by 6 protein peaks (m/z 2,759, 2,964, 2,047, 4,795, 4,139, and 37,761), which could distinguish local CRC patients (stage I and stage II) from regional CRC patients (stage III). By comparison, the serum biomarkers they found were quite different; this may be due to different types of chips they used and the patients included.

As serum protein profile alternates with the development of cancer, we deem that there must be some proteins representing the characteristic of lymph node involvement of CRC. Considering that IMAC30-Cu^2+^ chips are the improvement of IMAC3-Cu^2+^ chips and sporadic moderately differentiated adenocarcinoma accounts for about 50% of all CRC, we studied lymph node stage of the colorectal sporadic moderately differentiated adenocarcinoma using IMAC30-Cu^2+^ chips.

Also, we compared the sensitivity and specificity of the classification tree we built up and those of TRUS and MRI reported in lectures, the former surpass the later.

The present work explored a panel of highly sensitive and specific serum biomarkers using the SELDI protein chip technology, combining with an artificial intelligence classification algorithm. The model could classifiy node (+) patients of colon cancer and rectum cancer from the node (−) ones with a sensitivity close to 100%. These results suggested that the diagnosis ability of the model plays high sensitivity and specificity in classifying both colon cancer and rectum cancer. Although, these biomarkers provide a potential diagnostic platform for CRC node-staging, which need conformation and reproducibility by much larger and more detailed dataset, the point is that such an innovative clinical diagnostic method has the potential to improve the preoperative node-staging and optimize the individual management of CRC. The 22 protein peaks, either over- or low expressed in CRC with regional lymph node involvement, are now being identified by HPLC and MALDI-MS-MS in our laboratory.

In brief, the serum protein profiling using SELDI-TOF-MS could differentiate CRC with regional lymph node involvement from patients without regional lymph node involvement with a higher degree of sensitivity, specificity, and accuracy. This pioneering technology will doubtless enjoy a promising development room for figuring out CRC preoperative node-staging, efficiently and veraciously.

## Figures and Tables

**Table 1:** SELDI protein peak intensities in serum with significant differences (*P* < 0.05) between node-positive CRC patients and node-negative CRC patients.

Peak, m/z	Protein quantification ($\bar{x}\pm \text{SD}$)		*T*	*P*
	Node (+)	Node (−)		
5,867	3.534 ± 0.547	0.230 ± 0.107	58.749	0
9,290	3.271 ± 0.587	1.443 ± 0.103	73.932	0
7,970	12.364 ± 1.08	7.913 ± 0.668	61.235	1*E* − 10
14,433	0.891 ± 0.168	0.404 ± 0.102	42.436	1.8*E* − 09
15,621	1.782 ± 0.609	5.873 ± 0.655	23.125	2.16*E* − 08
15,824	0.729 ± 0.111	2.072 ± 0.255	20.218	4.25*E* − 08
13,652	1.388 ± 0.168	2.423 ± 0.259	26.276	7.58*E* − 07
3,781	4.268 ± 0.216	6.799 ± 0.977	33.346	9.28*E* − 07
15,006	0.641 ± 0.150	1.824 ± 0.084	19.552	2.26*E* − 06
7,802	6.580 ± 0.504	5.170 ± 0.635	16.319	5*E* − 05
4,497	9.154 ± 0.639	5.139 ± 0.916	22.740	0.000106
7,504	0.034 ± 0.005	0.704 ± 0.271	10.960	0.000106
5,967	8.809 ± 0.705	6.021 ± 0.879	18.793	0.000124
5,808	4.947 ± 0.797	2.252 ± 0.990	19.989	0.001097
10,057	1.809 ± 0.301	1.206 ± 0.245	8.139	0.001261
7,597	44.557 ± 1.121	37.993 ± 1.763	13.041	0.001549
5,786	16.269 ± 1.880	11.966 ± 0.994	10.461	0.003002
15,089	0.708 ± 0.198	1.678 ± 0.042	7.895	0.003002
3,104	4.719 ± 0.478	6.626 ± 0.791	7.436	0.012677
14,869	1.922 ± 0.653	4.278 ± 1.079	6.209	0.0134
7,620	12.534 ± 2.920	8.908 ± 1.474	13.036	0.017595
5,662	1.760 ± 0.601	0.266 ± 0.131	12.497	0.035988

Peaks were named by their mass to charge ratio (m/z).

**Table 2:** Important peaks selected by the biomarker pattern software.

Peak, m/z	Score*	
5,867	100.00	∣∣∣∣∣∣∣∣∣∣∣∣∣∣∣∣∣∣∣∣∣∣∣∣∣∣∣∣∣∣∣∣∣∣∣∣∣∣∣∣∣∣
9,290	100.00	∣∣∣∣∣∣∣∣∣∣∣∣∣∣∣∣∣∣∣∣∣∣∣∣∣∣∣∣∣∣∣∣∣∣∣∣∣∣∣∣∣∣
7,970	78.81	∣∣∣∣∣∣∣∣∣∣∣∣∣∣∣∣∣∣∣∣∣∣∣∣∣∣∣∣∣∣∣∣∣
15,823	67.55	∣∣∣∣∣∣∣∣∣∣∣∣∣∣∣∣∣∣∣∣∣∣∣∣∣∣∣∣
15,621	63.21	∣∣∣∣∣∣∣∣∣∣∣∣∣∣∣∣∣∣∣∣∣∣∣∣∣∣

Peaks were named by their mass to charge ratio (m/z).

*The most important peak was assigned an importance index of 100. The importance of other peaks was compared with the top peak and a number below 100 was given for each peak.

**Table 3:** Performance of the classification tree analysis of node-positive CRC patients in training set and test set.

Sets	Sensitivity (%)	Specificity (%)	Accuracy rate (%)
Training set			
Learning mode	100.00% (70/70)	100.00% (75/75)	100.00% (145/145)
Test mode	94.29% (66/70)	100.00% (75/75)	97.24% (141/145)
Test set	91.43% (32/35)	96.67% (29/30)	93.85% (61/65)

Number in parentheses denotes the number of correctly classified samples of the total number of samples in the group.
